# NO PENSION, NO HAPPINESS?—FINANCIAL SUPPORT EXPECTATION IN THE OLDEST-OLD AGE AND DEPRESSIVE SYMPTOMS

**DOI:** 10.1093/geroni/igad104.1651

**Published:** 2023-12-21

**Authors:** Chengming Han, Nan Zhou

**Affiliations:** Case Western Reserve University, Cleveland, Ohio, United States; Case Western Reserve University, Cleveland, Ohio, United States

## Abstract

**Objective:**

This paper aims to explore the effect of financial support expectation on depressive symptoms among the oldest-elderly. Method. Data were drawn from the China Health and Retirement Longitudinal Study (CHARLS) 2018. The analytical sample included 10641 respondents who were older than 45. Financial support expectations refer to whom they would reply on when they cannot work, which includes four categories: children, themselves (savings or commercial life insurance), pensions, and others. Linear regression models were employed after controlling for pension, health insurance and urban-rural household registration (hukou).

**Results:**

More than half of the sample reported that they would rely on their children when they became too old to work. Those who reported that they would rely on themselves (b=-1.72, p=0.000) or pensions (b=-1.23, p=0.000) reported lower levels of depressive symptoms compared to those who would rely on their children. When pension and health insurance were controlled for, only those who would rely on themselves presented lower level of depressive symptoms. Pension and health insurance (except rural health insurance) mediated the association between the financial support expectation and depressive symptoms. However, the hukou status inhibited the mediating effect of pension and health insurance.

**Conclusion:**

Financial uncertainty is a potential threat in the oldest-old age. Reducing the urban-rural inequality in pension and health insurance would help reduce the negative effects of financial uncertainty.

